# Improving transparency and quality assurance: Operationalising semi-automated data provenance tracking in a Trusted Research Environment

**DOI:** 10.23889/ijpds.v9i5.2540

**Published:** 2024-09-18

**Authors:** Babak Jahanshahi, Duncan McVicar, Neil Rowland

**Affiliations:** 1 Queen's Business School

## Objectives

This study used Census data from Northern Ireland linked to administrative data on prescriptions between 2010 and 2016, to examine the association between ambient air pollution exposure and the risk of Parkinson’s Disease (PD) onset. It contributes to this literature by providing new evidence in a comparatively low pollution context, using rich and nationally representative cohort data with comprehensive information on Parkinson’s Disease medications dispensed over an extended period.

## Approach

We estimated Cox Proportional Hazards models for the association between pollution exposure and PD onset as proxied by first receipt of PD medication, controlling extensively for potentially confounding factors at the neighbourhood, household, and individual levels. Estimates are presented in the form of hazard ratios for the effect of medium-term PM2.5 exposure on the risk of PD onset.

## Results

There was a clear unadjusted association between medium-term exposure to ambient pollution and the risk of developing PD, with those experiencing higher exposures being at greater risk. Once individual, household and neighbourhood confounders were adjusted for, however, we found no evidence supporting such associations. However, there was evidence of a positive association with both PM2.5 and NO2 exposure for sub-samples by age and education.

## Conclusions

This study contributes to an emerging literature examining the association between ambient PM2.5 pollution and onset of PD. Despite finding an unconditional association, we find no evidence for an association once individual, family and contextual characteristics are controlled for, at least in the relatively low-pollution context of Northern Ireland.

